# Advanced Imaging Modalities to Monitor for Cardiotoxicity

**DOI:** 10.1007/s11864-019-0672-z

**Published:** 2019-08-08

**Authors:** Andreas Seraphim, Mark Westwood, Anish N. Bhuva, Tom Crake, James C. Moon, Leon J. Menezes, Guy Lloyd, Arjun K. Ghosh, Sarah Slater, Heather Oakervee, Charlotte H. Manisty

**Affiliations:** 1 Department of Cardiovascular Imaging, Barts Heart Centre, Barts Health NHS Trust, West Smithfield, London, EC1A 7BE UK; 2 Institute of Cardiovascular Sciences, University College London, Chenies Mews, London, UK; 3 Department of Cardio-oncology, Barts Heart Centre, Barts Health NHS Trust, West Smithfield, London, EC1A 7BE UK; 4 Department of Haematology, Barts Health NHS Trust, West Smithfield, London, EC1A 7BE UK; 5 Department of Oncology, Barts Health NHS Trust, West Smithfield, London, EC1A 7BE UK

**Keywords:** Cardiotoxicity, Cardiac imaging, Chemotherapy, Cancer treatment, Cancer, Cardiac MRI, Echocardiography, Nuclear imaging

## Abstract

Early detection and treatment of cardiotoxicity from cancer therapies is key to preventing a rise in adverse cardiovascular outcomes in cancer patients. Over-diagnosis of cardiotoxicity in this context is however equally hazardous, leading to patients receiving suboptimal cancer treatment, thereby impacting cancer outcomes. Accurate screening therefore depends on the widespread availability of sensitive and reproducible biomarkers of cardiotoxicity, which can clearly discriminate early disease. Blood biomarkers are limited in cardiovascular disease and clinicians generally still use generic screening with ejection fraction, based on historical local expertise and resources. Recently, however, there has been growing recognition that simple measurement of left ventricular ejection fraction using 2D echocardiography may not be optimal for screening: diagnostic accuracy, reproducibility and feasibility are limited. Modern cancer therapies affect many myocardial pathways: inflammatory, fibrotic, metabolic, vascular and myocyte function, meaning that multiple biomarkers may be needed to track myocardial cardiotoxicity. Advanced imaging modalities including cardiovascular magnetic resonance (CMR), computed tomography (CT) and positron emission tomography (PET) add improved sensitivity and insights into the underlying pathophysiology, as well as the ability to screen for other cardiotoxicities including coronary artery, valve and pericardial diseases resulting from cancer treatment. Delivering screening for cardiotoxicity using advanced imaging modalities will however require a significant change in current clinical pathways, with incorporation of machine learning algorithms into imaging analysis fundamental to improving efficiency and precision. In the future, we should aspire to personalized rather than generic screening, based on a patient’s individual risk factors and the pathophysiological mechanisms of the cancer treatment they are receiving. We should aspire that progress in cardiooncology is able to track progress in oncology, and to ensure that the current ‘one size fits all’ approach to screening be obsolete in the very near future.

## Introduction

With more than 14 million new diagnoses of cancer in 2018 alone [1] and with half of patients expected to live for 10 years or more, the importance of healthy survivorship in oncology is increasingly important. Improved survival rates alongside the wealth of novel therapies prescribed to older patients with more co-morbidities have resulted in greater incidence of cardiac complications during cancer treatments, which then negatively impact cancer outcomes. Early detection and treatment of emergent cardiotoxicity has been shown to both reduce cardiovascular adverse events [2], and enable better treatment of the underlying cancer. However, over-cautious diagnosis and management from a cardiac perspective in this context may prevent patients from receiving optimal cancer treatment, thereby impacting on remission and survival rates.

Cardiooncology is a rapidly developing subspecialty within cardiology which aims to optimize diagnosis and management of cardiac complications of cancer treatment [3, 4]. Unfortunately our understanding of the underlying pathophysiology and natural history of cardiotoxicity remains limited, and it is generally only detected once cardiovascular disease presents clinically [5, 6]. Over the past decade, the armoury of anti-cancer therapies has expanded enormously [7], many of which are targeted therapies based on tumour genetic and receptor profiles, rather than simply tumour location. Unfortunately these are accompanied by a growing range of cardiovascular sequelae, including not only heart failure and left ventricular systolic dysfunction (cancer therapeutics–related cardiac dysfunction, CTRCD) but also myocarditis, arrhythmias, thrombotic events, coronary, pericardial and valvular heart disease. Recognition of these cardiovascular effects has led to screening being incorporated into both clinical pathways and research trials. However, how this is best achieved and with which imaging biomarkers and modalities remains controversial. Cardiac imaging has progressed significantly over the past two decades, and advanced echocardiographic techniques including three dimensional imaging, cardiovascular magnetic resonance (CMR) imaging, computed tomography (CT) and nuclear techniques including positron emission tomography (PET) have the potential to improve diagnostic accuracy of screening and offer an insight into the underlying pathophysiology of cardiotoxicity.

The decision to employ one imaging modality over another depends on several factors, including the technical and professional resources available, financial costs, patient preferences and modality-specific advantages and limitations (Table 1). An ideal imaging biomarker should be accurate and precise (with excellent inter-study reproducibility for serial screening), give an insight into the underlying pathophysiology and have sufficient sensitivity to detect disease before it becomes clinically apparent. This has been recognized in the context of cardiotoxicity for many years [8], but now more than ever the additional considerations of cost, availability and safety (lack of ionizing radiation) have governed decision making, rendering adoption of advanced imaging modalities in routine clinical workflows more challenging.

**Table 1:** Strengths and limitations of different imaging modalities for diagnosis and monitoring of cardiotoxicity

Imaging modality	Volume/function assessment	Tissue/mass characterisation	Myocarditis/inflammation	Valve disease	Pericardial disease	Coronary disease/ischaemia	Radiation exposure	Reproducibility/accuracy	Cost	Availability
2D echo	+	+	0	+++	++	0	None	+	+	+++
3D echo	++	++	0	+++	+	0	None	++	+	++
Stress echo	++	0	0	++	+	+++	None	++	++	++
CMR	+++ (*)	+++ (*)	+++	++	+++	+++	None	+++	+++	++
PET	++	++	+++	0	++	+++	+++	+++	+++	+
Nuclear	++	+	+	0	++	++	+++	++	+	++
CTCA	+	+	0	+	++	+++**	+/++	+++	++	++

2D echo, 2-dimensional echocardiography; 3D echo, 3-dimensional echocardiography; Stress echo, stress echocardiography; CMR, cardiac magnetic resonance; PET, positron emission tomography; Nuclear (includes SPECT, MUGA); CTCA, computed tomography coronary angiogram. +++, excellent diagnostic accuracy or features/ high cost; ++, intermediate diagnostic accuracy or features/ intermediate cost; +, reasonable diagnostic accuracy or features/low cost; 0, unable to diagnose. *Established gold standard. **CTCA is the only non-invasive test that provides anatomical information with regards to presence of coronary disease. All other modalities rely on functional assessment

The focus of this review is to give an overview on the use of advanced imaging modalities for cardiotoxicity monitoring, focusing on their diagnostic capabilities and limitations as well as their potential future applications.

## Monitoring for cancer treatment–related cardiac dysfunction

The cardiotoxic effects of anthracyclines and trastuzumab on cardiac function have long been recognized; however, many of the newer targeted therapies including tyrosine kinase inhibitors and immunotherapy are also associated with cardiac dysfunction. Baseline assessment of cardiac structure and function prior to initiating potentially cardiotoxic cancer treatments is essential, particularly in those at higher risk [9••, 10••, 11••, 12••, 13••, 14]. This is both an important component of initial risk assessment prior to start treatment, but also avoids inappropriately ascribing abnormalities detected during treatment to the therapy, in patients with pre-existing cardiomyopathies.

Left ventricular ejection fraction (LVEF) is the principal marker of left ventricular systolic function currently used both in clinical practice and research, and early asymptomatic declines are associated with subsequent progression to clinical heart failure in the context of cancer treatment [15–17]. Serial imaging is therefore recommended before, during (for HER2-targeted treatments) and on completion of treatment with anthracycline or other cardiotoxic agents. Cancer therapeutics–related cardiac dysfunction (CTRCD) has been defined as a drop in LVEF > 10% to below the lower limits of normal, although different absolute cut off values of abnormal LVEF are used [10••, 11••, 14, 18]. Reliable detection of CTRCD therefore depends not only on the sensitivity and accuracy of the imaging method to detect subtle changes in LVEF but also the ability to discriminate true changes in ejection fraction between studies from background noise (precision). This is determined by limitations in image quality and intra- and inter-observer reproducibility [19].

Echocardiography is recommended first line for cardiotoxicity screening by all of the current published oncology and cardiology guidelines [9••, 10••, 11••, 12••, 13••, 14]. There are inherent advantages to the technique: low-cost, widespread availability, lack of ionizing radiation and patient acceptability; however, the accuracy of 2D echocardiography is limited by its reliance on geometric assumptions and adequate acoustic windows (potentially worse in cancer patients, for example post-mastectomy). Test-retest variability in LVEF measurement by 2D echocardiography is however up to 10% [19, 20] and it has been questioned whether it can reliably detect the 5–10% change used to define CTRCD [21]. The use of transpulmonary contrast results in a higher level of precision than 2D alone, especially when windows are limited [22] and forms part of international LV assessment guidelines [23]. 3D echocardiography is a more precise method for measurement of LV volume and function against a gold standard of CMR [24–27]. Unfortunately, however, it is feasible in only 60% of patients post-anthracycline chemotherapy for breast cancer [28], due to poor echocardiographic windows in this population

Multigated acquisition (MUGA) scans [29] have historically been widely used for LVEF evaluation, and were the imaging modality of choice in clinical trials of anthracycline cardiotoxicity in the 1980s due to widespread availability [15]. Despite good intra- and inter-observer reproducibility [30], measures of LVEF by MUGA are only modestly accurate when compared with a gold standard of CMR, with misclassification of 35% of subjects when cardiotoxicity was diagnosed using a LVEF threshold of 50% [31]. MUGA is further limited by both the associated radiation exposure, and the limited information it provides on other cardiac structures. Single-photon emission computed tomography (SPECT) enables acquisition of 3D images and provides an additional option for LVEF evaluation. It can provide information on right ventricle function and wall motion abnormalities; however, it tends to underestimate LVEF values compared to MUGA and echocardiography [32]. Positron emission tomography (PET) is unlikely to have a widespread role in screening for cardiotoxicity despite its accuracy [33], because of high cost and radiation exposure along with limited availability.

CMR imaging is now the gold standard for evaluation of ventricular volumes and function [34], with proven superior reproducibility for LVEF assessment [35]. LVEF is calculated from a stack of short axis cine images of the heart, with the endocardial borders segmented either manually or automatically at end-diastole and end-systole, in order to provide the cavity areas for each slice. Summation of the slice areas enables calculation of the LV end-diastolic and end-systolic volume, from which ejection fraction can be calculated. With temporal variability in LVEF measurements estimated at 2.4 to 7.3% [20, 36] and without the constraints of reliance on acoustic windows, CMR is well-suited for monitoring for cardiotoxicity, particularly in those whose echocardiographic images are suboptimal [37]. The enhanced reproducibility of CMR over echocardiography for detecting small changes in LVEF translates into the potential for smaller sample sizes in clinical studies. CMR-derived measures of LVEF are now commonly used as the endpoint in randomized trials evaluating the value of cardioprotective agents for prevention of cardiotoxicity [38, 39]. Importantly, CMR also provides additional information on LV structure parameters such as LV mass, which has been shown to independently predict cardiovascular events in patients following anthracycline therapy [40]. Although both access to and costs of CMR have historically limited its use, rapid CMR protocols [41], 10 or 20 min, can be adapted to cardiooncology, enabling cheaper, shorter scans that can deliver improved efficiency.

## Imaging biomarkers of early subclinical CTRCD

Management of heart failure secondary to cancer therapies can be challenging if diagnosed late, with prognosis historically worse with anthracycline-related cardiomyopathy than other aetiologies of heart failure [42]. By the time a drop in LVEF is detected, the opportunity for maximal therapeutic intervention may have already been missed [43]. Data from 2625 patients followed during and after treatment with anthracyclines showed that the incidence of cardiotoxicity was 9%, with 98% of cases arising within the first year following treatment, of whom the majority had at least partial recovery if treatment was started early [44]. Alongside this, histological data has found significant myocellular injury on biopsy despite preserved ejection fraction, suggesting that LVEF may be a relatively late marker of cardiotoxicity [45].

Although echocardiographic markers of diastolic function including tissue Doppler velocities have been explored in early cardiotoxicity, studies show conflicting results [46, 47] [48]. Stress imaging has also been explored as a potential tool for early detection of cardiotoxicity [49]. Stress echocardiography was shown to detect subclinical cardiac dysfunction in young adults treated with anthracyclines [50], but the incremental value of stress echocardiography and the role of contractile reserve in cardiotoxicity monitoring remains unclear [51, 52]. Myocardial deformation using left ventricular (LV) strain, strain rate and twist [53–55] by echocardiography are more sensitive and earlier biomarkers of cardiotoxicity than LVEF, and permit detection of cardiotoxicity at lower chemotherapy doses than were historically believed to be associated with cardiac damage [56]. A relative reduction of peak LV systolic global longitudinal strain (GLS) by 10 to 15% is an early predictor of subsequent cardiotoxicity [57–59], and 3D GLS may detect cardiotoxicity earlier than 2D GLS [60, 61]. The ongoing SUCCOUR trial [62] will be the first randomized controlled study using GLS as a predictive biomarker for CTRCD, and the results will likely impact clinical practice.

More recently, CMR-derived GLS (using feature tracking) has been shown to detect LV dysfunction before LVEF falls [63] and to be an independent predictor of all-cause mortality across all cardiomyopathies [64]. Reductions in both global circumferential and longitudinal strain have been demonstrated in patients receiving doxorubicin and trastuzumab, which correlated with changes in subclinical declines in LVEF [65–67], highlighting its potential use for monitoring of early cardiotoxicity from chemotherapy.

## Imaging biomarkers to understand the pathophysiology of CTRCD

Left ventricular dysfunction is the most frequent final manifestation of cardiotoxicity, but may result from a variety of different, treatment-specific, pathophysiological mechanisms [68] including myocyte apoptosis [69], myocardial fibrosis, inflammation [70] and ischaemia [71]. An ideal imaging biomarker would interrogate individual pathways directly, to detect cardiotoxicity prior to the development of myocardial mechanical dysfunction. Furthermore, the introduction of new therapies targeting different treatment pathways, including novel immunomodulatory strategies such as adoptive T cell therapy (ACT) and immune checkpoint inhibitors (ICI), has resulted in new and less well-defined mechanisms of injury to the heart, often resulting in a wide spectrum of toxicity and clinical presentations, ranging from asymptomatic detection of elevated cardiac biomarkers to cardiogenic shock [72, 73]. Advanced imaging offers the potential for tissue characterisation, and hence the ability to detect myocardial oedema and inflammation, focal and diffuse fibrosis and assess myocardial perfusion.

**Fig. 1:**
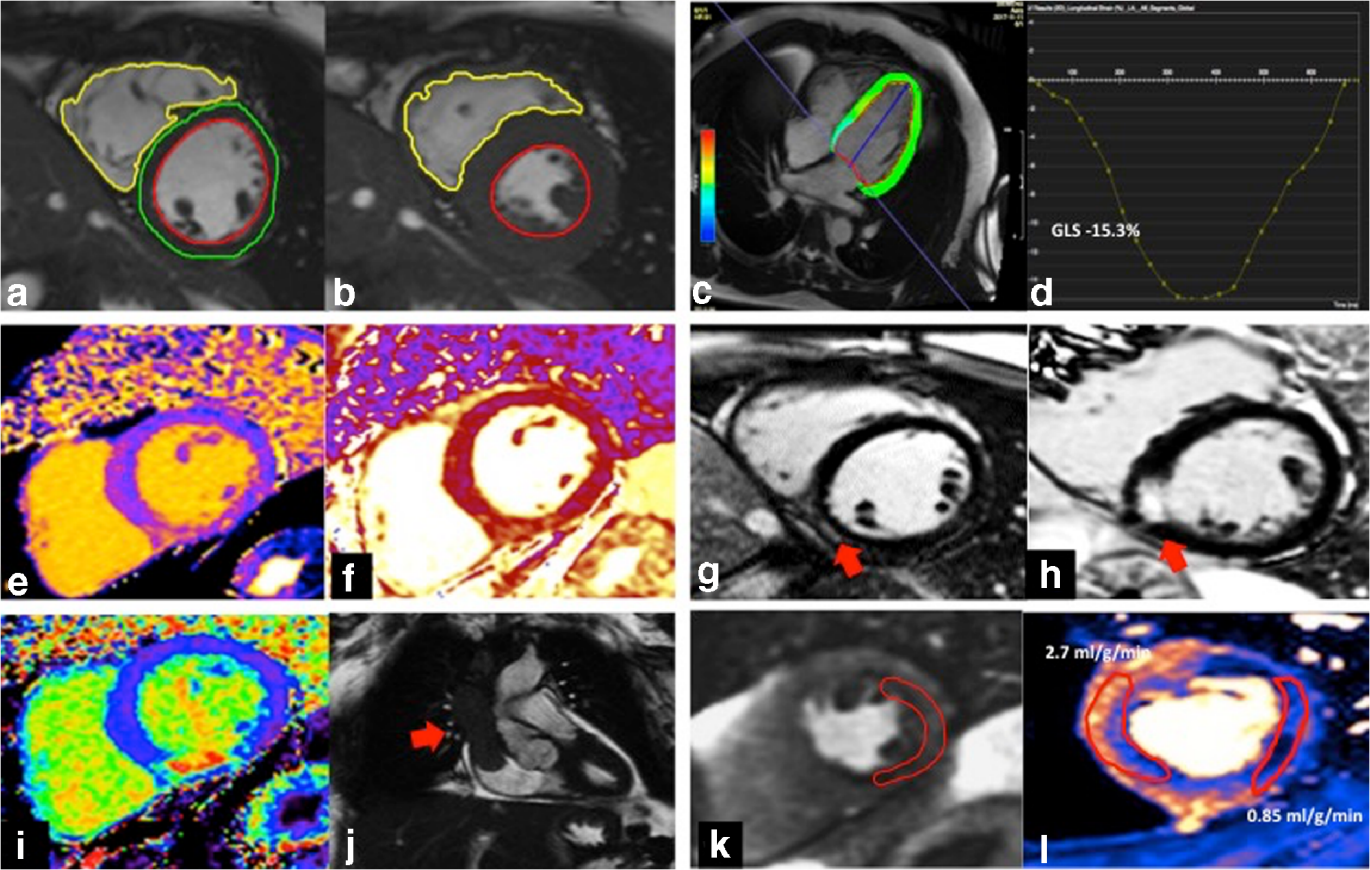
Multiparametric cardiac evaluation using CMR. **a–d** Cardiac function and volumetric assessment: Endocardial and epicardial borders are drawn in diastole (**a**) and systole (**b**) allowing calculation of LVEF, chamber volumes and myocardial mass. Measurement of myocardial deformation using feature tracking CMR (**c**) enables measurement of global longitudinal strain (GLS), a marker of early, subclinical cardiotoxicity (**d**)**. e–j** Tissue characterisation: Quantitative parametric mapping techniques such as T1 (**e**), T2 (**f**) and extracellular volume (ECV) mapping , **(i)** detect and measure diffuse myocardial fibrosis and oedema. Late gadolinium enhancement imaging **(g,h)** identifies focal fibrosis, and can differentiate between underling pathologies such as myocarditis (subepicardial, **g**) and infarction (subendocardial, **h**). Intracardiac masses (**j**, arrow), here in the superior vena cava and the right atrium, can be identified and tissue characterisation enables stratification of the underlying tissue type**. k–l** Myocardial perfusion imaging can assess for functionally significant coronary artery disease and aid risk stratification pre-cancer treatment or surgery. Here, a region of relative hypo-perfusion (**k**, outlined myocardium) is seen with vasodilator stress, with reduced myocardial blood flow at 0.85 ml/g/min demonstrated on the quantitative perfusion map **(l)**, related to circumflex territory ischaemia.

CMR adds value because of its myocardial tissue characterisation capabilities with growing evidence within cardiooncology (Fig. 1). Gadolinium-based contrast agents can be administered to detect focal myocardial scarring and fibrosis, and the distribution and extent of myocardial scar can be used both to differentiate aetiologies of myocardial disease (for example ischaemic cardiomyopathy versus myocarditis) and to estimate prognosis [74, 75]. Although experimental models of anthracycline cardiotoxicity [76] demonstrated focal scarring on late gadolinium enhancement (LGE) imaging, clinical studies suggest LGE is rarely detected, and is not associated with outcomes [77, 78]. Rather than focal scarring, anthracyclines are thought to cause diffuse interstitial fibrosis, via excess collagen deposition. Diffuse fibrosis can be quantified by CMR using pre- and post-contrast T1 mapping, and this technique has been validated against biopsy-measured collagen volume fraction with good reproducibility across a spectrum of other cardiac disorders [79, 80]. T1 mapping has been explored as a biomarker of early anthracycline cardiotoxicity, with several small studies showing elevated myocardial T1 and extracellular volume fraction (ECV) in patients treated with anthracyclines compared with age- and sex-matched controls [81, 82]. Other studies have however failed to reproduce these results [78, 83], particularly in lower risk patients. This conflicting data therefore means that, at least currently, T1 mapping is not a tool for anthracycline- or HER2-related mainstream cardiotoxicity screening.

CMR can also detect myocardial oedema and inflammation using T2-weighted imaging sequences, with higher T2 relaxation times seen with increasing myocardial water content. Parametric mapping techniques have been developed to quantify T2 relaxation times, and recent data from a pig model of doxorubicin-induced cardiotoxicity using serial multiparametric (T1, T2 and ECV) mapping has shown that T2 values provide the earliest marker of myocardial damage [84•]. Myocardial T2 values increased early following administration of anthracyclines, correlating with increase myocardial water content, despite normal T1 and ECV values and no detectable abnormalities in myocardial contractility. On stopping anthracycline administration when T2 levels increased, T2 values normalized and LV dysfunction did not progress, suggesting that cardiotoxicity may be largely reversible when detected at this early stage. Albeit from animal data with intra-coronary chemotherapy injection, this study provides evidence that T2 imaging may be a potential early imaging biomarker for cardiotoxicity with anthracyclines. T1 and T2 mapping techniques are also useful for the diagnosis and monitoring of myocarditis, which has been associated with various cancer agents, with increasing recognition in the context of immunotherapies [85]. Although there is insufficient data currently available to clarify the role of CMR in immunotherapy-related myocarditis, it is likely that the ability of this modality to detect both oedema and fibrosis will deliver clinical utility.

PET imaging can detect alterations in myocardial metabolism and inflammation [86], offering good sensitivity for the diagnosis of myocarditis and therefore potentially other cardiotoxicities. PET data [87] suggests that changes in myocardial glucose metabolism can be detected early in the course of anthracycline treatment, with low baseline myocardial ^18^F-FDG uptake predicting a progressive increase in cardiac glucose consumption during and after chemotherapy, as well as a higher incidence of cardiotoxicity [88].

## Evaluation of coronary artery disease and myocardial ischaemia

Whilst cardiotoxicity related to cancer therapy generally focuses on left ventricular impairment [89], cancer treatments can cause other clinical cardiac syndromes including coronary events, pericardial disease, valvular heart disease, pulmonary hypertension and right ventricular dysfunction [90–94] (Table 2).

**Table 2:** Types of cancer therapy and their associated cardiotoxicity risk

Cancer therapy	LVD	Myocarditis	HTN	Valve disease	Coronary spasm	Coronary thrombosis	Atherosclerosis	Pericardial constriction	Pericardial effusions	Pulmonary HTN	QT -prolongation	Arrhythmia	Conduction disease
Anthracyclines	+++								+			+	
HER2 Monoclonal Antibodies	+++												
VEGF monoclonal antibodies	++		+++			++							
BCR-ABL tyrosine kinase inhibitors	++*	++*		++*			++*	++*	++*				
VEGF tyrosine kinase inhibitors	++		+++			++					+++		
Bruton kinase inhibitor			++									++	
Immune checkpoint inhibitors	++	++											
Proteasome inhibitors	++	++	+++			++				++			
Fluoropyrimidines					+++	++						+	
Arsenic trioxide									+++		+++		
Alkylating agents	++					++			+	+			
All-transretinoic acid									++				
Immunomodulatory drugs (myeloma)	++					++						++	
Radiotherapy (mantle/high dose)	++		+++	+++			+++	++					++
Radiotherapy (low dose)			++	++			++						

The risk assigned to each treatment represents an overview of the overall risk associated with that class of chemotherapy treatment; however, drug-specific risk within each category may lie outside the range listed. +++, treatment associated with > 10% risk of developing that form of cardiotoxicity; ++ risk is estimated to be between 1% and 10%; +, risk estimated to be < 1%; *, drug-dependent risk; LVD (left ventricular dysfunction); HTN (hypertension); CAD (coronary artery disease)

Several cancer treatments have been implicated in the development of myocardial ischaemia and coronary events including myocardial infarction, including fluoropyrimidines, platinum compounds, VEGF inhibitors, certain bcr-abl tyrosine kinase inhibitors and radiotherapy [10••] (Table 2). Pathophysiological mechanisms differ by drug and include accelerated atherosclerosis, coronary spasm, vascular endothelial damage and arterial thrombotic events. Most cardiac imaging modalities including echocardiography, CMR and nuclear imaging can be used for functional testing in this context, with exercise or stress agents including adenosine, regadenoson and dobutamine used to unmask ischaemia or myocardial perfusion abnormalities. There are small differences in the diagnostic performance of these tests [95]; however, the selection of an individual modality is generally based on local expertise and availability. CMR can sensitively detect myocardial infarction using LGE, and quantitative myocardial perfusion mapping offers the potential to directly quantify regional myocardial perfusion reserve [96], previously only feasible with nuclear imaging techniques. CT coronary angiography provides a non-invasive anatomical assessment of coronary artery disease, and with an excellent negative predictive value can offer a reliable test for exclusion of significant coronary disease and for risk stratification prior to surgery or administration of the drugs listed above.

## Valvular heart disease

Valve disease is a rare complication of chemotherapy, however is well-recognized as a late consequence of high-dose radiotherapy to the mediastinum (particularly with historical techniques such as mantle field radiotherapy). There is a latent interval of 10–20 years between radiation exposure and development of clinically significant heart valve disease, with risk related both to radiation dose and interval from exposure, with reported prevalence rates of 5–32% in patients treated for Hodgkins lymphoma [97]**.** Importantly, surgical outcomes in these patients are worse than in a matched cohort of patients undergoing valve replacement [98], meaning that early detection and accurate assessment is critical, with non-surgical, percutaneous valve implantation approaches playing an increasing role [99].

Echocardiography remains both the first-line and gold standard imaging modality for functional assessment of valvular heart disease, allowing qualitative and quantitative evaluation of both stenotic and regurgitant valves. Computed tomography and CMR [100] can also be used for valve evaluation, with the former being useful for stenotic valve planimetry and evaluation of suspected endocarditis [101], particularly when hybrid imaging such as PET-CT is employed [102]. CMR can measure flow across valves using phase contrast imaging, and hence is often employed where echocardiographic assessments are of poor quality or uncertain [103].

## Pericardial disease

Pericardial disease is a common finding with cancer therapy, and pericarditis, pericardial effusion and constrictive pericarditis are all seen associated with both chemotherapy agents (including anthracyclines, cytarabine, arsenic and tyrosine kinase inhibitors), and mediastinal radiotherapy [104]. Pericarditis can arise acutely during radiotherapy, leading to later pericardial constriction which typically presents over 10 years following treatment and has a cumulative incidence of up to 5% in this population [10••]. Echocardiography is the first-line imaging modality for pericardial assessment, including diagnosis and functional characterisation of constrictive and tamponade physiology, whilst CT is able to reliably detect the pericardial calcification generally seen as a late complication of radiotherapy. CMR offers additional diagnostic information, combining both sensitive structural imaging (using dark blood T1 weighted imaging with and without fat saturation) with tissue characterisation (multiparametric mapping and LGE) and functional assessment (real-time cine imaging during free breathing) for more detailed investigation of pericardial disease [105]. This can be particularly useful where echocardiography is inconclusive, or where more detailed tissue characterisation is required. Formal diagnosis and evaluation of constrictive pericarditis can be challenging, and a clinical role remains for invasive cardiac catheterisation with haemodynamic assessment in some circumstances.

## Pulmonary hypertension

Although a rare complication of cancer therapy, development of pulmonary hypertension has been observed with dasanitib (Bruton’s kinase inhibitor used in chronic myeloid leukaemia where prevalence of pulmonary hypertension (PAH) is 5%), cyclophosphomide and other alkylating agents [106, 107]. Echocardiography is preferred as the initial imaging modality with repeated assessment every 3–6 months recommended in patients receiving PAH-associated therapy [10••].

## Cardiac masses

Whilst echocardiography is generally the initial modality to detect cardiac masses (and is best for small, rapidly-moving masses including valve vegetations), assessing for tissue invasion and for differentiating mass aetiology generally requires advanced imaging techniques. CT has the spatial resolution to accurately determine the location, size and relationship of the mass to tissue planes, but CMR plays a key role [108, 109] in helping evaluate between different types of mass due to its inherent tissue characterisation sequences. After locating a mass on dark and bright blood sequences, T1- and T2-weighted imaging techniques, early and late gadolinium imaging and rest perfusion imaging can help determine the aetiology and potential resectability of the mass. Thrombi (common on indwelling venous catheters) can be easily detected using early gadolinium imaging, and malignant tumours are more likely to have heterogeneous signal intensity, cross tissue planes, and enhance on LGE and rest perfusion imaging [110]. Whilst a definitive malignant tissue diagnosis is rarely possible, key benign aetiologies can be detected (cysts, lipoma, thrombus). PET/CT imaging also has a role in differentiating benign from malignant tumours, and for detecting cardiac metastases [111] although careful patient preparation is key to obtaining diagnostic cardiac imaging.

## Light chain cardiac amyloidosis

Patients with myeloma and other haematological malignancies rarely develop light chain amyloidosis, which may present with cardiac amyloidosis—an infiltrative cardiomyopathy, often presenting as heart failure with preserved ejection fraction (HFPEF). The diagnosis is often suspected from the characteristic echocardiographic appearances of left ventricular hypertrophy, preserved LV function, impaired RV function and profound apical sparing on strain maps [112]. Endomyocardial biopsy, whilst a definitive test if positive, carries inherent risks due to its invasive nature, meaning that a non-invasive diagnostic test is needed. CMR findings in cardiac amyloid are characteristic and can be used as prognostic markers, with left ventricular hypertrophy, abnormal gadolinium kinetics, subendocardial or transmural late gadolinium enhancement and significantly elevated myocardial T1 and ECV levels often detected in patients with cardiac involvement [113]. Nuclear bone scintigraphy techniques (using ^99m^Tc-PYP and ^99m^Tc-DPD) can help differentiate light chain (AL) from transthyretin amyloidosis and a novel PET radiotracer (18F-florbetapir) [114] has recently been shown to be of potential value for detecting and quantifying AL amyloid in the heart.

## Conclusions

Improved outcomes in oncology mean that it is increasingly important to prevent, detect and treat any early signs of treatment-related cardiotoxicity so patients can receive optimal cancer treatment but minimize subsequent cardiovascular morbidity and mortality. Advanced cardiac imaging techniques offer more sensitive and reproducible screening options than conventional 2D echocardiography or MUGA, and may provide novel insights into the underlying pathophysiology of CTRCD. The development of novel cancer treatments is currently rapid, and often cardiotoxicity is not detected until after initial phase 1 and 2 safety trials [115], with the causative mechanisms poorly understood. The need for accurate cardiac imaging biomarkers is therefore greater than ever, meaning that provision and access to CT, CMR and nuclear imaging will require expansion to match the growing demand from cardiooncology.
